# Case report: Corpectomy and iliac crest bone autograft as treatment for a vertebral plasma cell tumor in a dachshund dog

**DOI:** 10.3389/fvets.2023.1281063

**Published:** 2023-12-11

**Authors:** Hannah R. Yoder, Megan R. MacRae, Olivia M. Snead, Karl H. Kraus

**Affiliations:** Veterinary Clinical Sciences, College of Veterinary Medicine, Iowa State University, Ames, IA, United States

**Keywords:** ilium, autograft, corpectomy, plasma cell, multiple myeloma, case report

## Abstract

A 7-year-old, 7.5 kg, female spayed dachshund dog presented to a veterinary teaching hospital after being referred for lameness and the inability to urinate. The dog presented as weakly ambulatory paraparetic with intact pain perception. Computed tomographic (CT) imaging showed ventral bony lysis and periosteal proliferation of the L3 vertebra, consistent with a plasma cell tumor. A corpectomy of the L3 vertebra was performed and subsequently stabilized with autogenous cortico-cancellous iliac crest bone staves, autogenous cancellous bone, and bilaterally placed locking plates [String of Pearls (SOP^®^), Orthomed]. The dog recovered well, with no decrease in neurologic status overnight, and continued to improve until discharge. Upon a recheck exam at 4 weeks postoperatively, the dog appeared neurologically improved with only mild ambulatory proprioceptive ataxia of the hind limbs. This case demonstrates that the transposition of a non-vascularized iliac crest autogenous bone graft with stabilization via SOP^®^ plates and screws can be used in the management of dogs with vertebral plasma cell tumors and should be considered as a surgical option in similarly affected cases.

## 1 Introduction

Vertebral plasma cell tumors account for 3%–6% of all vertebral tumors in dogs and may exist as a solitary plasmacytoma or as the disseminated disease, multiple myeloma, with the former reported to have a better prognosis (1, 2). In humans, 50% of solitary plasmacytomas later develop into disseminated diseases (3). Vertebral plasma cell tumors may involve more than one vertebra, sometimes producing multifocal signs due to compression of the spinal cord and secondary vertebral instability. These signs may include spinal hyperesthesia and proprioceptive deficits (4, 5). Diagnosis of vertebral plasma cell tumors is assisted by spinal radiography, CT myelography, and MRI (6). Radiography may be used as a screening tool for vertebral neoplasms. Typical changes include discrete, well-defined lytic lesions without sclerosis of the surrounding bone (7). CT is considered the imaging modality of choice due to its high specificity in detecting lytic osseous lesions, usefulness in the staging process, and ability to identify the extent of bone involvement (8). Hypercalcemia in serum biochemistry is often seen in conjunction with bone lysis on diagnostic imaging (6). Diagnosis of multiple myeloma in contrast to plasmacytoma in dogs requires two or more of the following criteria to be met: radiographic evidence of osteolytic lesions; a bone marrow biopsy with greater than 5% plasma cells; monoclonal gammopathy in the serum or urine; or light chain proteinuria (9).

Surgical excision via corpectomy is proposed for some primary vertebral tumors and the debulking of malignant tumors (5). During a corpectomy, the affected portion of the vertebral body is removed. The excised bone may be replaced with either a spacer fashioned from polymethylmethacrylate or a graft that is then anchored by plate constructs to stabilize the spinal cord (2). Literature cites freeze-dried femoral allografts, ilium, rib, and coccygeal vertebra as possible sources of bone grafts (2, 10–12). In human neurosurgery, iliac crest autographs are used in anterior cervical spine surgery and are reported to have the highest fusion and lowest complication rates compared to other grafts (13). Stabilization of the unstabilized spine following debulking of a vertebral neoplasm in humans is considered palliative, regardless of the extent of neoplastic disease. This is attributed to the location, unresectability, and metastatic potential of vertebral tumors (14).

This report aims to describe the successful use of an iliac crest bone autograft after the excision of a vertebral plasma cell tumor via corpectomy in a dog.

## 2 Case presentation

### 2.1 Clinical history

A 7-year-old, 7.5 kg spayed female dachshund with a body condition score of 5/9 presented to Iowa State University Emergency Services with a 1-day history of paraparesis and urinary retention. Three weeks prior, the dog was treated by the referring veterinarian for back pain and a rigid abdomen with prednisone (0.6 mg/kg orally q24h), gabapentin (5 mg/kg orally q8-12h, increased to 10 mg/kg orally q8-12h), and methocarbamol (1.5 mg/kg orally q12h). Prior to referral, the patient had been in good health.

### 2.2 Physical exam

A neurologic examination revealed mild hind limb paresis when walking and lumbar spinal hyperesthesia upon palpation. The clinical examination was otherwise unremarkable. The dog was treated with gabapentin (10 mg/kg orally q8-12h), prednisone (0.6 mg/kg orally q24h for 5 days, then 0.6 mg/kg q48h for 10 days), and the recommendation of 4–6 weeks of strict cage rest. Despite these treatments, 4 days later, the dog presented to Iowa State University Orthopedic Surgery for the inability to stand the previous day. A neurologic examination revealed decreased proprioceptive reflexes in the pelvic limbs, which were more exaggerated on the left side, mild hyperreflexia of the pelvic limb reflexes, spinal hyperesthesia in the lumbar region, and pain upon the palpation of the iliopsoas muscle. The patient was noted to have a modified Frankel score of 2. The dog was able to stand and urinate at this time. A complete blood count (CBC), serum biochemistry profile, and SDMA did not reveal any abnormalities. A urinalysis revealed proteinuria of 3+ and a urine specific gravity of 1.024. A urine protein:creatinine ratio (UPC) of 9.34 indicated high levels of protein compared to creatinine, confirming high levels of protein in the patient’s urine unrelated to urine concentration. Protein electrophoresis of the urine revealed albumin as the source of protein in the urine. A distinct monoclonal peak to suggest Bence–Jones proteinuria was not appreciated.

### 2.3 Diagnostic imaging

**Figure 1:**
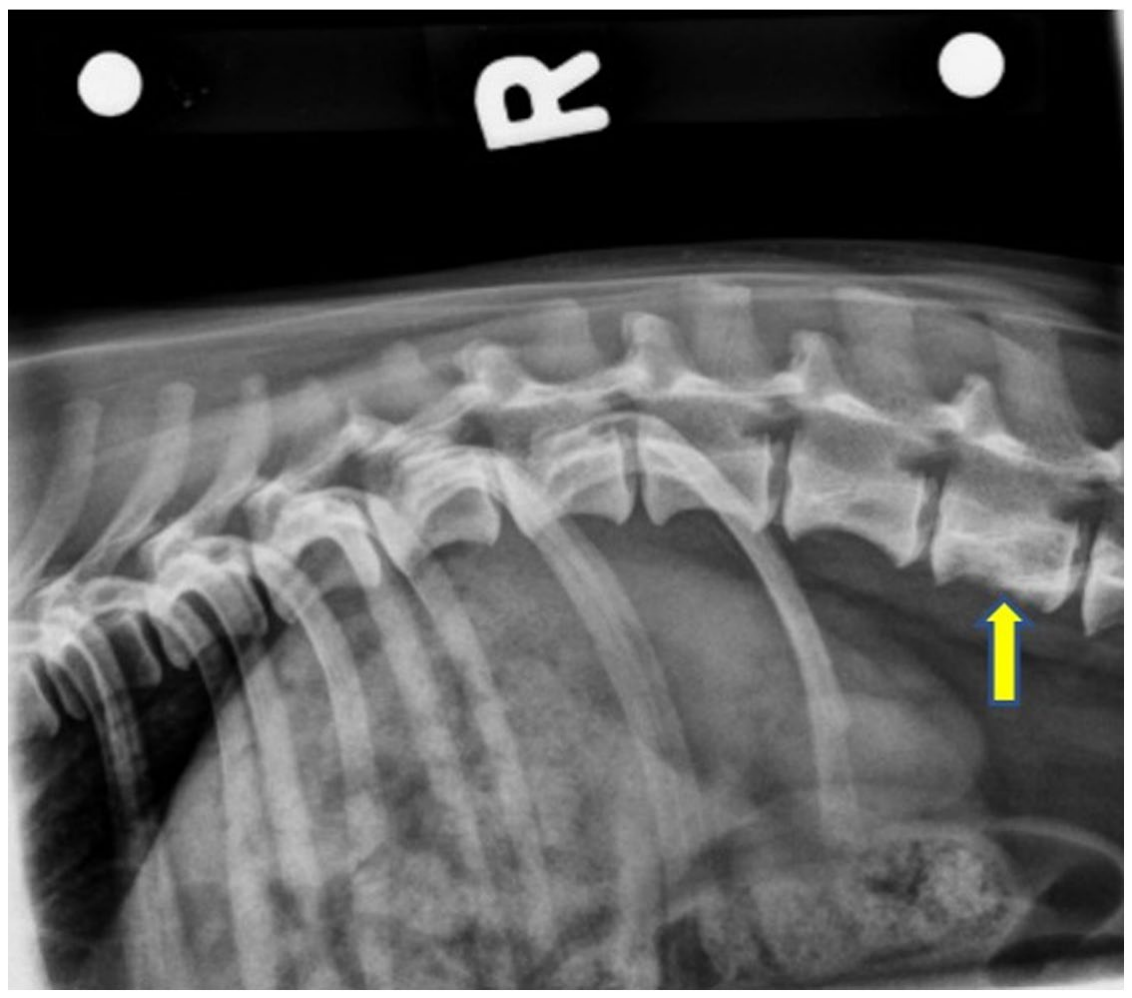
Right lateral pre-operative radiograph showing smooth periosteal proliferation from the ventral margin of the L3 vertebral body, as well as an ill-defined increase in mineral opacity centrally. The L3 vertebral body has a mildly moth-eaten lysis caudally. The yellow arrow indicates L3.

**Figure 2:**
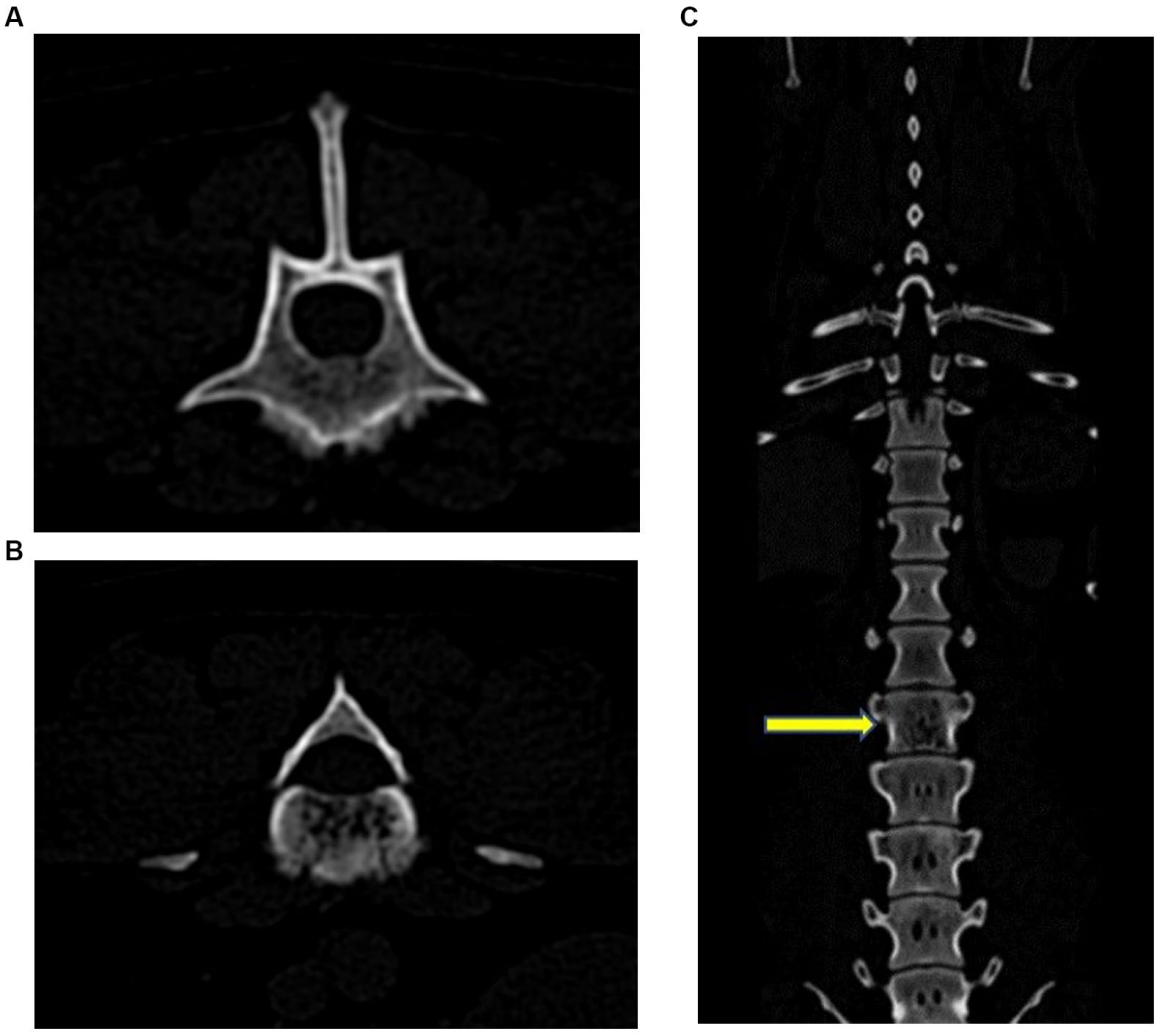
Transverse **(A)** and **(B)**, and coronal **(C)** CT images. There were multiple punctate hypoattenuating defects within the L3 vertebral body that coalesced caudally into a zone of moth-eaten lysis. There was a fluffy periosteal reaction ventrally, with multiple focal regions of cortical loss. The yellow arrow indicates L3.

Radiographs of the thoracolumbar spine revealed narrowing of the T11–T12 intervertebral space relative to adjacent disk spaces. No mineralized disk material was identified. There was a smooth periosteal reaction from the ventral margin of the L3 vertebral body, as well as a poorly marginated increase in mineral opacity centrally that was consistent with sclerosis. The L3 vertebral body had mildly moth-eaten lysis, and the lateral margins of the spinal canal were poorly marginated. The lysis did not affect the lamina or pedicle of the vertebra (Figure 1). A computed tomography (CT) scan was performed using a 16-slice Canon Aquilion scanner (TSX201A, Canon Medical Systems, Tustin, California). Contiguous images were reconstructed in transverse, coronal, and sagittal planes using bone and soft tissue algorithms before and after IV administration of 15 mL of iohexol positive contrast (Omnipaque, Penn Veterinary Supply Inc.). The images showed multiple punctate hypoattenuating defects within the L3 vertebral body that coalesced caudally into a zone of moth-eaten lysis. There was a fluffy periosteal reaction ventrally, with multiple focal regions of cortical loss. On the post-contrast scan, there was a long, crescent-shaped zone of contrast enhancement within the ventral spinal canal, dorsal to L3, causing mild extradural compression of the cord (Figure 2). The next day, the dog was placed under general anesthesia for a bone biopsy of the L3 vertebra. Two fine needle aspirate (FNA) samples of the L3 vertebra were obtained under fluoroscopic guidance and submitted for cytology. A Jamshidi needle was used to obtain three bone biopsies from the body of the L3 vertebra after first confirming the location via a wire guide and fluoroscopy imaging. The bone biopsies revealed a variable mix of mature and reactive bones. On histopathology, a cellular mass was not observed, and a neoplastic process could not be confirmed.

### 2.4 Surgical procedure

**Figure 3:**
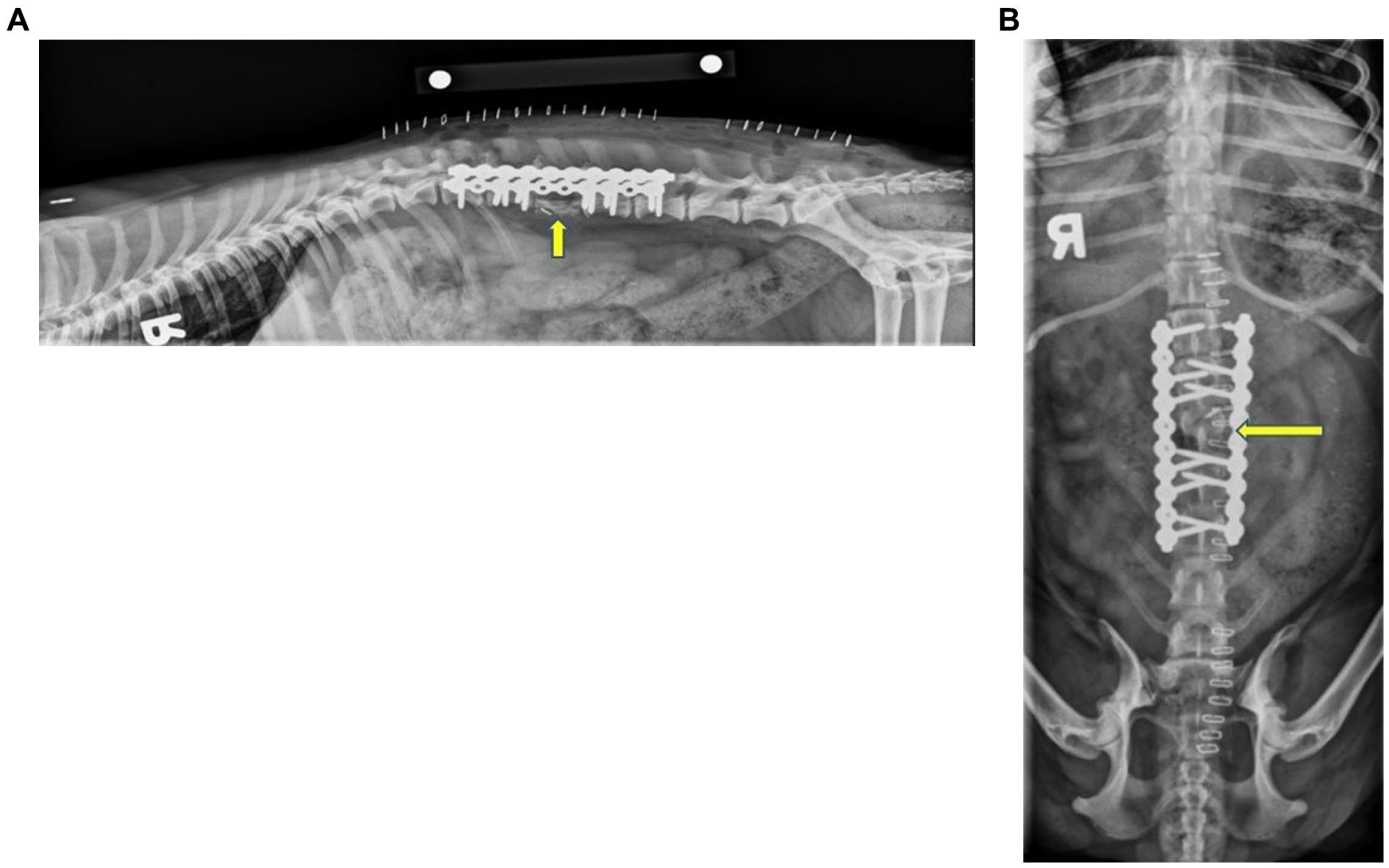
Immediate postoperative right lateral **(A)** and ventrodorsal **(B)** radiographs of the thoracolumbar spine. The images show that the two 10-hole String of Pearls (SOP^®^) plates were applied to the right and left dorsolateral aspects of the vertebral bodies. Note that the first screw on the left plate did not engage the cortex of L1. This configuration resulted in one screw engaging the body of L1, four in the bodies of L2 and L4, and two screws engaging the body of L5. The articular facets and spinous process of L3 remained in the appropriate location and were static. A metallic hemoclip was superimposed over the spinal canal on the ventrodorsal projection at the level of cranial L3. There was an irregularly shaped, curvilinear new bone where the body of L3 was removed. The dorsal aspect of the iliac wings was irregular with flattened irregular margins, consistent with ostectomy. The yellow arrow indicates L3.

Surgery to remove the affected vertebral body was performed 3 days following diagnostic imaging. The dog was placed under general anesthesia according to the anesthesiologist’s preference, prepared for surgery, and positioned into sternal recumbency. An incision was made just dorsal to the wings of the ilium over the left iliac crest, and a blunt dissection was performed down to the level of the craniodorsal iliac spine to collect an autogenous bone graft. Soft tissues, fibrous tissues, and the fibrocartilage of the iliac crest were removed. An oscillating saw was used to cut the wing of the ilium into 5 mm strips. Thereafter, a bone curette was used to remove cancellous bone from the shaft of the ilium. The iliac crest cortico-cancellous bone staves and cancellous bone grafts were saved for use later in the procedure. The incision was lavaged, and the hypodermal layer was closed with 3–0 polydioxanone (PDS, Ethicon) in a simple continuous pattern along with skin staples. This procedure was then repeated contralaterally. Once the grafts were retrieved, the vertebral and costal landmarks were palpated, and a standard dorsal approach to the lumbar spine was performed. The incision began at the level of T11 and extended to the level of L6, approximately 14 cm in length. Hemostasis was maintained with bipolar electrocautery and hemostats. A #15 scalpel blade and Metzenbaum scissors were used to incise through the subcutaneous tissues down to the level of the dorsal spinous processes, starting along the midline and moving along the right side of the dorsal spinous processes. The right epaxial muscles were elevated from the underlying bone with a Freer periosteal elevator to expose the lamina, articular facets, and vertebral pedicles. The epaxial muscle attachments to the articular facets in this region were removed with curved Mayo scissors and bipolar electrocautery. Gelpi retractors were used to maximize exposure to the surgical site. The location was verified by the palpation of the 13th rib and the transverse process of L1. A pneumatic-powered handpiece (Surgairtome Two-Powered Drill, Hall) was then used to incise the left L3 pedicle, ventral to the facets. Soft tissue dissection was made lateral and ventral to the L3 vertebra, attempting to maintain a fascial layer outside of the vertebral mass. The annulus fibrosis of the L2 and L4 intervertebral disks was sharply incised with a scalpel from the adjacent disk endplates. The body of L3 was exposed from the level of the facet ventrally and as far across the midline as possible. Rongeurs were used to remove the remainder of the pedicle, transverse process, annulus fibrosis, and body of the vertebra. These tissues were submitted for histopathology. A ball-tipped probe was used to remove the endosteum and palpate the spinal cord. Mild venous sinus oozing occurred and was addressed with intermittent suction. Hemostasis was achieved with cautery, ligation, and hemostatic clips. After as much of the vertebral pedicle and body were removed from the left side, the endplates of the defect were scarified with a pneumatic burr. A 10-hole 2.7 mm String of Pearls (SOP®) plate was used to stabilize the left side. One screw engaged the L1 vertebral body, two engaged the L2 vertebral body, two screws engaged the L4 vertebral body, and one engaged the L5 vertebral body. The procedure was repeated on the right side, completely removing the vertebra from the level of the facet ventrally and keeping as much soft tissue as possible. A second plate was placed on the right with identical screw placement, resulting in a total of six screws proximal and six screws distal to the removed L3 vertebra. The left and right surgical sites were lavaged thoroughly, and the surgical team changed gloves and instrumentation. Thereafter, the sections of the iliac crest staves were cut to length and placed into the defect from the left and right sides. A total of five stave pieces were used. The cancellous bone graft was then packed into any defects around the cortico-cancellous staves. The dorsal spinal fascia was closed with 3–0 polydioxanone (PDS, Ethicon) in a simple continuous pattern. The skin was closed with 3–0 poliglecaprone (Monocryl, Ethicon) in an intradermal suture pattern overlaid with staples. Postoperative lateral and dorsoventral radiographs were performed and showed screw placement and positioning of the bone graft, as shown in Figure 3. The most proximal screw was noted to not engage the body of L1. The remainder of the screws appropriately engaged the vertebral bodies. The dorsal aspect of the ilial wings was noted to be flattened and irregular, which was consistent with ostectomy. The patient recovered uneventfully and was transferred to the ICU for overnight monitoring.

### 2.5 Postoperative care

The patient recovered from anesthesia uneventfully and was ambulatory paraparetic the following day. The patient was assigned an MFS of 2 at this time. Two days postoperatively, the dog was transferred to ISU’s rehabilitation service. Upon discharge, 6 days postoperatively, the dog was ambulatory with no obvious ataxia. The neurologic examination at the time of discharge was considered within normal limits, with only mild spinal hyperesthesia present and an MFS of 1. The histopathologic findings were received 2 weeks following surgery and were consistent with a plasma cell neoplasm. Chemotherapy was discussed with the owners at this time but was declined due to the patient’s lack of clinical signs.

### 2.6 Recheck exam

**Figure 4:**
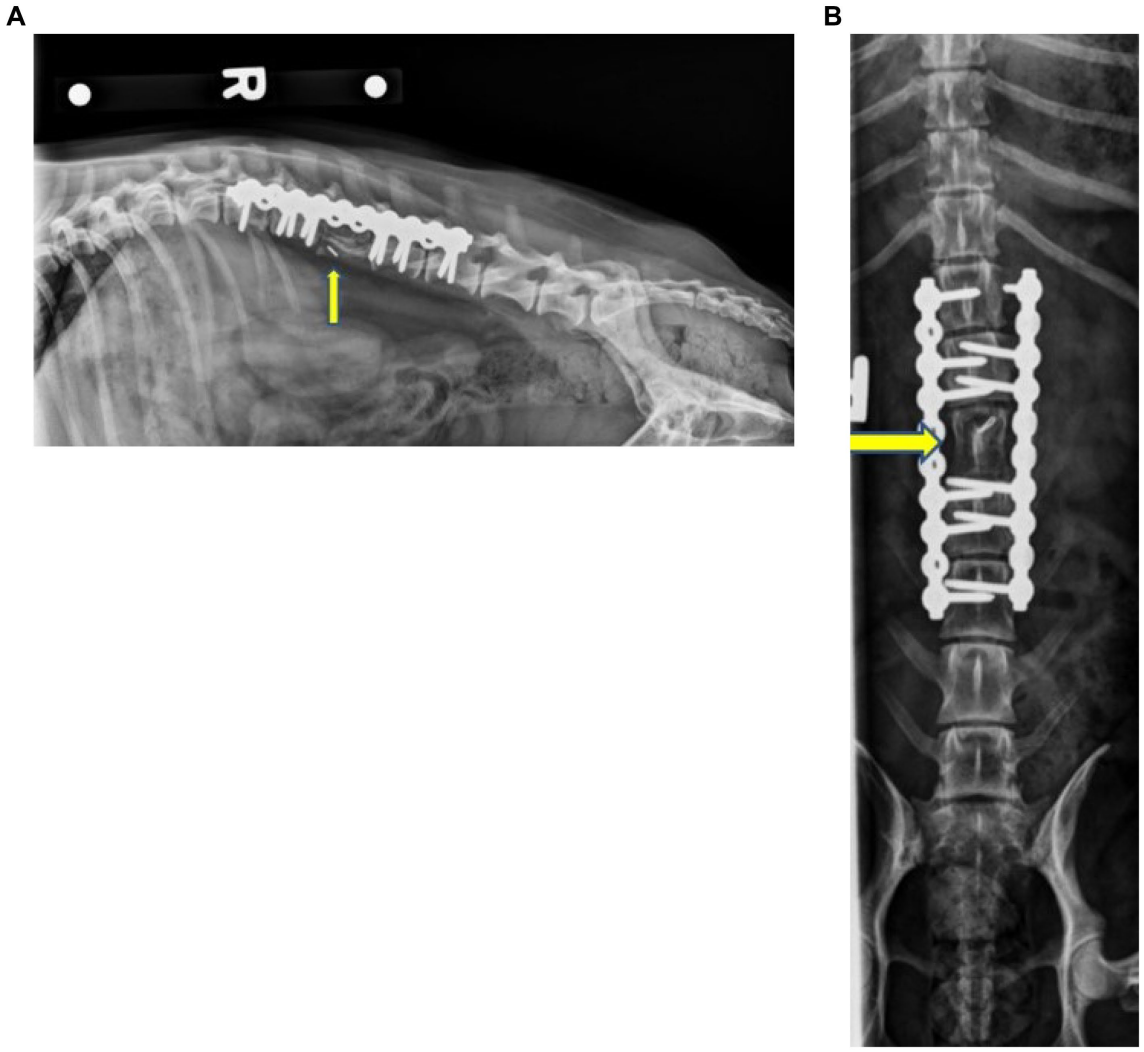
Right lateral **(A)** and ventrodorsal **(B)** radiographs of the thoracolumbar spine 2 months postoperative showing the static L3 corpectomy with the stabilization of the spine using two SOP^®^ plates and associated screws. No evidence of implant failure/loosening was noted. The mineral opacity in the location of L3, as well as the ostectomy of the iliac wings, was consistent with a remodeling bone graft. The yellow arrows indicate L3.

On follow-up examination 1 month after surgery, the dog was walking with only mild ambulatory proprioceptive ataxia of the hind limbs and an MFS of 2. The patient was urinating and defecating normally when observed. Two months following surgery, the dog presented to Iowa State University Emergency Services due to generalized abdominal or lumbar pain and discomfort that increased in intensity over 2 weeks. The dog was clinically within normal limits and was placed on fluids and pain management. The dog was transferred to Iowa State University Oncological Services for further assessment. Spinal radiographs showed no evidence of implant failure or loosening at this time, and no evidence of bone lysis was appreciated. Osseous integration of the cortico-cancellous autograft was noted at this time (Figure 4). No abnormalities were attributed to the graft or implant at this time. The dog was sent home with Gabapentin (6.6 mg/kg PO q 8–12 h), Metacam (0.2 mg/kg PO q 24 h), and a fentanyl patch (25mcg). The dog was noted to improve with this conservative management. The dog presented to another veterinarian 4 months after surgery due to abdominal pain. Serum biochemistry showed hypoalbuminemia, borderline hypoglycemia, elevated ALP, azotemia, and mild anemia. CT and radiographs were recommended at this time, but the owners opted for humane euthanasia.

## 3 Discussion

Solitary vertebral plasma cell tumors rarely occur in dogs, accounting for only 3%–6% of all vertebral tumors (1, 2). Their rarity does not undermine their significance, however, since solitary plasmacytomas of bone may represent an early stage of multiple myeloma (2). To the best of the authors’ knowledge, this case report is the first to describe the use of a cortico-cancellous bone graft taken from the iliac crest following a corpectomy to remove a vertebral plasma cell tumor in a dog. Previously reported cases have used free fat grafts or cellulose membranes to fill bony defects following corpectomy (15–18). However, free-fat grafts have been associated with neurologic deficits and spinal cord compression (19). Cellulose mesh products may reduce epidural hematoma formation; however, these products have been found to cause extensive scar tissue that adheres to the dura mater and nerve roots (20). Iliac allografts have been reported but were not used in this case due to the lack of direct incorporation properties via osteoconduction compared to the more complete Haversian remodeling, incorporation, and osteogenesis of autografts (9, 21). Although autogenous cancellous bone is still considered the pre-eminent graft material, autografting may be limited by the amount of retrievable cancellous bone (22). The proposed advantage of using an iliac bone graft is that cortical bone autografts maintain structural strength and confer complete histocompatibility while possessing osteogenic healing potentials (23). The fully differentiated osteoblasts and undifferentiated stem cells that compose the iliac autograft, combined with the matrix and signals provided by the bone morsels, yield a mixture that leads to reproducible bone formation when placed into the surgical site (21). Additionally, the graft incorporation rates and time to fusion are generally considered excellent for the iliac crest (24).

In this case, chemotherapy was not initiated because there were no signs of systemic or local disease and the blood work was within normal limits at the 2-month recheck. At this time, the implants were stable, and integration of the cortico-cancellous bone staves was noted. In this case, the SOP® bone plates were used to prevent the risk of fixation failure. The String of Pearls plate was used in this study due to its locking plate construct, which has been associated with decreased implant failure (25). Additionally, the SOP® plate has been noted to have greater ultimate and failure loads when compared to veterinary acetabular plates or screw/wire/polymethylmethacrylate constructs (26).

## 4 Conclusion

This report details the management of a dog with a vertebral plasma cell tumor, where the defect following corpectomy was filled with a non-vascular iliac crest bone autograft. We found that it was relatively easy to access the iliac crest, and the shape of the bone was deemed to be suitable for the purpose. Postoperative imaging confirmed the plate and screws remained radiographically unchanged and stable a month following the surgery. Although the dog experienced clinical signs of deterioration 2 months after the surgery, these symptoms were associated with severe GI signs. In conclusion, we have reported the surgical management of a vertebral plasma cell tumor of the L3 vertebra. Transposition of a non-vascularized iliac autogenous bone graft stabilized with a SOP^®^ locking plate and screws can be considered a surgical option in similarly affected cases.

The limitations of this report include the single case and its retrospective nature. Further prospective studies and biomechanical evaluations of this technique are needed to corroborate what this case demonstrated. Additionally, evaluation of the bone interface of the autografts and defect sites after surgery via CT could provide more in-depth results. Potential shortcomings of this approach include the cost associated with the diagnostic and surgical procedures, the dependence on patient size, and the risk of iatrogenic damage to the spinal cord.

## Data availability statement

The original contributions presented in the study are included in the article/supplementary material, further inquiries can be directed to the corresponding author.

## Ethics statement

Ethical review and approval was not required for the study involving animals in accordance with the local legislation and institutional requirements. The owner of this patient had signed a general consent form to release the details of this case for use in research as the patient was referred to a veterinary school teaching hospital.

## Author contributions

HY: Writing – original draft. MM: Writing – review & editing. OS: Writing – review & editing. KK: Writing – review & editing.
